# Improved R2* liver iron concentration assessment using a novel fuzzy c-mean clustering scheme

**DOI:** 10.1186/s12880-015-0097-5

**Published:** 2015-11-03

**Authors:** Pairash Saiviroonporn, Vip Viprakasit, Rungroj Krittayaphong

**Affiliations:** Division of Diagnostic Radiology, Department of Radiology, Faculty of Medicine Siriraj Hospital, Mahidol University, Bangkok, 10700 Thailand; Haematology/Oncology Division, Department of Pediatrics and Thalassemia Center, Mahidol University, Bangkok, Thailand; Division of Cardiology, Department of Medicine, Faculty of Medicine Siriraj Hospital, Mahidol University, Bangkok, Thailand

**Keywords:** FCM, Liver iron overload, Liver R2* measurement, Liver segmentation, Thalassemia

## Abstract

**Background:**

In thalassemia patients, R2* liver iron concentration (LIC) measurement is a common clinical tool for assessing iron overload and for determining necessary chelator dose and evaluating its efficacy. Despite the importance of accurate LIC measurement, existing methods suffer from LIC variability, especially at the severe iron overload range due to inclusion of vessel parts in LIC calculation. In this study, we build upon previous Fuzzy C-Mean (FCM) clustering work to formulate a scheme with superior performance in segmenting vessel pixels from the parenchyma. Our method (MIX-FCM) combines our novel 2D-FCM with the existing 1D-FCM algorithm. This study further assessed possible optimal clustering parameters (OP scheme) and proposed a semi-automatic (SA) scheme for routine clinical application.

**Methods:**

Segmentation of liver parenchyma and vessels was performed on T2* images and their LIC maps in 196 studies from 147 thalassemia major patients. We used manual segmentation as the reference. 1D-FCM clustering was performed on the acquired image alone and 2D-FCM used both the acquired image and its LIC data. To execute the MIX-FCM method, the best outcome (OP-MIX-FCM) was selected from the aforementioned methods and was compared to the SA-MIX-FCM scheme. We used the percent value of the normalized interquartile range (*nIQR*) to its median to evaluate the variability of all methods.

**Results:**

2D-FCM clustering is more effective than 1D-FCM clustering at the severe overload range only, but inferior for other ranges (where 1D-FCM provides suitable results). This complementary performance between the two methods allows MIX-FCM to improve results for all ranges. OP-MIX-FCM clustering error was 2.1 ± 2.3 %, compared with 10.3 ± 9.9 % and 7.0 ± 11.9 % from 1D- and 2D-FCM clustering, respectively. SA-MIX-FCM result was comparable to OP-MIX-FCM result, with both schemes showing ability to decrease overall *nIQR* by approximately 30 %.

**Conclusion:**

Our proposed 2D-FCM algorithm is not as superior to 1D-FCM as hypothesized. In contrast, our MIX-FCM method benefits from the best of both methods to obtain the highest segmentation accuracy at all ranges. Moreover, segmentation accuracy of the practical scheme (SA-MIX-FCM) is comparable to segmentation accuracy of the reference scheme (OP-MIX-FCM). Finally, we confirmed that segmentation is crucial to improving LIC assessments, especially at the severe iron overload range.

## Background

Thalassemia major patients require lifelong regular blood transfusions, which cause tissue iron overload and can lead to serious complications if excess iron from the transfusions is not properly managed [1, 2]. Magnetic resonance imaging (MRI), which measures transverse relaxation rates (R2*), has become a convenient clinical tool to assess and monitor iron overload, especially in the heart and liver [3–6]. In a clinical setting, relaxivity measurements from the liver are converted based on biopsy results into liver iron concentration (LIC) data [7–10], which can then be used to determine necessary chelator dose and evaluate its efficacy [11–14]. As such, longitudinal iron overload studies benefit greatly from reliable and precise LIC measurements.

Liver R2* measurement consists of two stages: MR acquisition, which is well established, and post-processing analysis, which consists of various implementation methods without a standard dominant approach. Differences among analysis techniques include fitting together of criteria and models, and selection of region of interest (ROI) [15, 16]. Nevertheless, intra- and inter-observer variability has been reduced using an analysis method described previously [15, 17]. The method employs a median R2* calculated using pixel-wise criteria [5, 15, 17] with a constant-offset mono-exponential (C-EXP) model [18]. The ROI is selected as the entire liver with the obvious major vessels excluded, but some unavoidable components still remain as part of the LIC calculation. Such inclusion increases reported LIC variation, especially at the high LIC level due to considerable difference between the R2* of the vessels and the heavily overloaded parenchyma. Exclusion of such vessel parts from the calculation should, therefore, be greatly desired in the monitoring the chelator efficacy, especially at the high iron overload range [14, 17]. A posteriori segmentation of vessel pixels from the parenchyma can yield more robust estimates of LIC distribution and can further reduce reported LIC variation. Manual segmentation, however, is a time-consuming process and is prone to inter-observer variability. Therefore, automatic (without any user interaction) or semi-automatic (partial user interaction) segmentation is preferred, especially for routine clinical tasks.

Several such segmentation methods have been suggested by employing T2* thresholding [19–21] or image segmentation method [17, 22]. The latter method is based on Fuzzy C-Mean (FCM) clustering concept, which is suitable for clinical tasks because multiple clusters can be automatically assigned for each data element, increasing tolerance for variations and noise [23–26]. However, intensity inhomogeneity (bias field) can still cause erroneous segmentation and overlapping tissue classes. Various methods to minimize the bias field problem have been suggested [27–29], generally including an additional feature using smooth non-parametric gain fields.

Positano et al. [17] investigated FCM-based segmentation to improve liver R2* assessment. Their results showed the advanced FCM algorithm with gain-field correction on the acquired intensity (AI) images to provide the best outcome (100 % exclusion of vessels and 70 % inclusion of the parenchyma), as compared with various alternative algorithms. However, the advanced algorithm still relies on hardware information (e.g., effects of RF-coil-induced MR intensity inhomogeneity), limiting the general applicability of this approach. Thus, an algorithm providing a similar outcome without relying on such specific information would be preferable.

In this study, we propose a new 2D-FCM clustering algorithm using both AI and LIC to segment vessels from parenchyma based on the conventional FCM algorithm without any gain field models. Our method is based on the hypothesis that LIC data should improve clustering result because its value depends mainly on iron concentration, thus reducing bias field effects on AI images. The effect of which should facilitate implementation for generalized use in a clinical setting. Moreover, the 2D-FCM method should improve clustering accuracy, as compared with using only AI data (1D-FCM). The MIX-FCM method, which derives its results from the best possible clustering outcome from both 1D- and 2D-FCM results, was also evaluated. This study also assessed possible optimal clustering parameters (OP scheme) and proposes a semi-automatic (SA) scheme for routine clinical use.

## Design and methods

### Study group

The study used liver T2* MR images and LIC maps corresponding to 196 studies from 147 thalassemia major patients (60 males and 87 females; aged 20.3 ± 11.4 years) performed during the March 2009 to July 2011 study period. The protocol for this study was approved by the Siriraj Institutional Review Board (SIRB), Faculty of Medicine Siriraj Hospital (Si708/2014). Written informed consent was obtained from adult participants and from a parent or guardian of pediatric patients. R2* measurement results were grouped according to LIC levels [30] tabulated from biopsy results, as reported by Wood, et al. [9], as follows: normal (LIC < 3 mg/g dw, n = 27), mild (3 mg/g dw < LIC < 7 mg/g dw, n = 38), moderate (7 mg/g dw < LIC < 15 mg/g dw, n = 40), and severe (LIC > 15 mg/g dw, n = 91). To facilitate the investigation of clinically overloaded livers only, normal LIC-level cases were excluded, leaving a total of 169 studies in the investigation.

### MRI and interpretation

Liver MR images were performed on a 1.5 T Achieva-XR Quasar-Dual-Gradient System (Philips Medical Systems, Best, The Netherlands) using a five-element cardiac phase-array coil. We used a common method of liver R2* measurement, as described in previous reports [15, 31]. In brief, the acquisition part included a breath-hold multi-echo fast gradient-recalled-echo sequence using 20 echo times (TEs = 1.07-16.30 ms with 0.8 ms increments) from a mid-hepatic slice. The analysis phase consisted of pixel-wise fitting using the C-EXP model [5, 15, 22]. R2* values were calculated within a large manually-defined ROI of the entire liver parenchyma, excluding the major blood vessels [15, 22, 31]. R2* results were then calculated to LIC values, which were then reported as median and interquartile range (IQR) [9, 31]. To evaluate variability of the LIC measurement, *nIQR* was determined by normalizing IQR to its median:

$$ nIQR\left(\%\right)={\scriptscriptstyle \frac{\left({Q}_3-{Q}_1\right)\ast 100}{median}} $$

Where *Q*_1_ and *Q*_3_ represent the first and third quartiles of LIC data inside the ROI.

### Segmentation of liver parenchyma and cessels

Segmentation of vessels from parenchyma pixels was performed only inside user-defined ROIs. MATLAB Toolbox (MathWorks, Inc., Natick, MA, USA) was used for all analytical operations. Manual segmentation was based on thresholding and basic morphological operations by a medical expert on a TE image with a high-contrast signal between the parenchyma and vessels, as well as the corresponding LIC map. These manual segmentation results served as reference (ground truth) data for evaluation and comparison against all FCM clustering methods.

FCM is a pixel-wise iterative algorithm that aims to partition data by minimizing an objective function. The standard FCM objective function [23, 25] for segmenting input data of *n* pixels into *C* clusters is:

$$ {J}_m\left(u,v\right) = {\displaystyle \sum_{i=1}^C}{\displaystyle \sum_{j=1}^n}{u}_{ij}^m\ {d}^2\left({x}_j,{v}_i\right)\ \mathrm{subject}\ \mathrm{t}\mathrm{o}\ {\displaystyle \sum_{i=1}^C}{u}_{ij}=1\  and\kern0.5em {u}_{ij}\ \in\ \left[0,1\right] $$

Where *d*^2^(*x*_*j*_, *v*_*i*_) is the Euclidean distance (‖*x*_*j*_ − *v*_*i*_‖^2^) between data point *x*_*j*_ and centroid *v*_*i*_ of the *i*^*th*^ cluster; and *u*_*ij*_ is the membership of data *x*_*j*_ in the *i*^*th*^ cluster. The parameter *m* is the fuzzy factor, which controls the fuzziness of partition results (*m* ≥ 1). Furthermore, if allow *X* to be a *k* × *n* input data matrix, then *k* is the dimension of the feature vectors (i.e., dimension of input data) for each *x*_*j*_ and *n* is the number of feature vectors (i.e., number of image pixels).

**Fig. 1:**
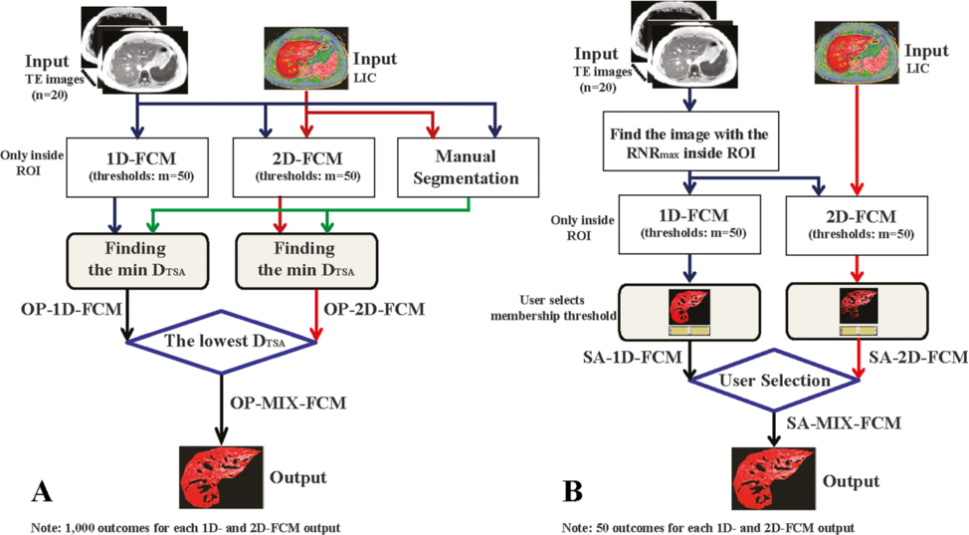
Diagrams of OP (**a**) and SA (**b**) schemes. Optimum (OP) scheme defines the best segmentation result from all possible clustering outcomes, as compared to manual segmentation. Semi-automatic (SA) scheme defines a TE input automatically from its RNR_max_ and allows user to manually select the best outcome and an appropriate membership threshold from pre-calculated images of both FCM types

We used FCM to segment the parenchyma and vessels (*C* = 2) and set *m* to the commonly used value of 2 [27]. There were two types of input data investigated. One involved only AI values (*k* = 1, 1D-FCM) and the other used both AI values and the corresponding LIC map (*k* = 2, 2D-FCM), as shown in Fig. 1a. Therefore, the input data were *x*_*j*_ = (*x*_*j*,*TE*_) and *x*_*j*_ = (*x*_*j*,*TE*_, *x*_*j*,*LIC*_) for 1D- and 2D-FCM, respectively, where *j* was a 2D image coordinate and *TE* and *LIC* denoted the AI value of the TE image and its LIC value, respectively. For both input data types, FCM outcomes were dependent on the selected TE image. Clustering results also depended on the defuzzified (membership) threshold (*u*_0_^2^), which assigns each pixel to a specific cluster. Therefore, to determine the optimum TE and threshold (OP scheme), we applied fifty threshold levels between 0 and 0.98 with 0.02 increments. As a result, OP scheme selects the best outcome or the most accurate clustering result (as compared to manual segmentation) from a total of 1,000 (i.e., 20 TEs and 50 *u*_0_^2^) FCM outcomes for each data input from both 1D- and 2D-FMC results (Fig. 1a). Moreover, the MIX-FCM method was also evaluated based on selection of the best clustering outcome from both results.

To quantitatively validate FCM clustering results compared to manual segmentation (ground truth), we employed tissue segmentation accuracy (*TSA*) [26], based on the dice coefficient [24], defined as follows:

$$ TSA\left(\%\right)=\frac{2{N}_{csi}\ast 100}{N_{tsi}+{N}_{tri}}\  or = \frac{TP\ast 100}{\left(\left(FP+TP\right)+\left(TP+FN\right)\right)/2} $$

Where *N*_*csi*_ denotes the number of pixels that are correctly assigned to tissue *i* by FCM clustering and *N*_*tsi*_ and *N*_*tri*_ are the total number of pixels assigned to tissue *i* in the clustering result and the reference (manual segmentation), respectively. Thus, *TSA* percentage is the set of true-positives (*TP*) divided by the average of the size of the clustering result (false-positives (*FP*) + *TP*) and the size of the ground truth set (false-negatives (*FN*) + *TP*). To further quantitatively delineate clustering results from both the parenchyma and vessels, we defined a *D*_*TSA*_ value, which quantifies proximity of FCM segmentation to the perfect outcome (ground truth), as follows:

$$ {D}_{TSA}\left(\%\right) = \sqrt{{\left(100-TS{A}_{liver}\right)}^2+{\left(100-TS{A}_{vessels}\right)}^2} $$

The lower the *D*_*TSA*_ value, the more accurate the FCM clustering result (i.e., perfect clustering corresponds to *D*_*TSA*_ = 0). The OP scheme for the 1D- and 2D-FCM methods identifies the best outcome from the lowest of the two *D*_*TSA*_ values, which are derived from all possible TE input and membership thresholds. The OP-MIX-FCM scheme, on the other hand, identifies the result from the lowest *D*_*TSA*_ value of these two methods (Fig. 1a).

In addition, a semi-automatic (SA) scheme (Fig. 1b), which aims for a practical implementation, was also proposed and investigated. In the SA scheme, one FCM variable (TE input) was selected automatically, while the other (membership threshold) was chosen manually by the user. We propose selection of one variable (as opposed to two) to be more practical for the user in practical clinical application. The scheme calculates the signal range to noise ratio (RNR), which is defined as the difference between the largest and smallest signal intensity inside the ROI to the standard deviation of noise signal from each TE image. The TE input to the SA-1D- and SA-2D-FCM calculations is then chosen from the image with the maximum RNR (RNR_max_). The MIX-FCM method is also used in this scheme. The user, therefore, needs to choose the best outcome and membership thresholds (which have the same number of levels as in the OP scheme) from the pre-calculated images of both FCM types. Consequently, there will be 50 outcomes (50 *u*_0_^2^) for each SA-1D- and SA-2D-FMC output from which the user may select. The SA-MIX-FCM scheme, thus, serves as a practical implementation alternative, while the OP-MIX-FCM scheme serves best as the optimal outcome or reference alternative.

### Statistical analysis

MATLAB Statistics Toolbox was used for all statistical analysis. Paired Student’s *t*-test and Mann–Whitney *U* test were used for parametric and non-parametric evaluations between two data sets of *D*_*TSA*_ and *nIQR*, respectively. For all statistical analysis, *p* < 0.05 was considered as the threshold for statistical significance. Coefficient of variation (CV), signifying level of agreement, was calculated from the mean (bias) ± 1.96 standard deviation (SD) of the differences between each method pair and then divided by their mean, resulting in a percentage. CV was used to evaluate levels of agreement of *nIQR* between manual-segmentation and all FCM-segmentation results.

## Results

In this study, FCM clustering from the parenchyma was found to be more accurate than from the vessels (Table 1). The 2D-FCM method was significantly more accurate than the1D-FCM method for vessel segmentation only, while MIX-FCM yielded a superior result to both individual methods in both anatomical clusters. For both OP and SA schemes, vessel segmentation accuracy (*TSA*) of 2D-FCM clustering was significantly better than that of 1D-FCM (*t*-test: *p* = 0.004 and *p* < 0.001, respectively), but there was no significant difference in the parenchyma (*t*-test: *p* = 0.384 and *p* = 0.142, respectively). MIX-FCM clustering of both schemes was far superior (*t*-test: *p* < 0.001) to that of its individual component methods for both segmentation outputs. Moreover, *TSA* results from both OP-MIX-FCM and SA-MIX-FCM schemes were not statistically different (*t*-test: *p* < 0.001) for both anatomical clusters. Thus, the MIX-FCM algorithm improves segmentation accuracy, compared with using either the proposed method or the existing method alone. In addition, segmentation accuracy of the practical scheme (SA-MIX-FCM) was comparable to that of the reference scheme (OP-MIX-FCM).

**Table 1:** Mean ± SD of *TSA* values

Method	OP scheme		SA scheme	
	Parenchyma (%)	Vessels (%)	Parenchyma (%)	Vessels (%)
1D-FCM	98.3 ± 1.7	89.8 ± 9.8	96.9 ± 2.3	81.7 ± 11.0
2D-FCM	98.6 ± 3.3	93.2 ± 11.4	97.6 ± 4.8	88.9 ± 13.9
MIX-FCM	99.7 ± 0.4	97.9 ± 2.3	98.8 ± 1.1	92.9 ± 5.5

Mean ± SD of *TSA* values from parenchyma and vessel classes of 1D-, 2D-, and MIX-FCM clustering for both OP and SA schemes. This shows the 2D-FCM method to be significantly more accurate than 1D-FCM for vessel segmentation only, while MIX-FCM method yields a superior result over both individual component methods and for both anatomic segmentations

From Table 2, the segmentation error (*D*_*TSA*_) from the OP and SA schemes of the MIX-FCM method for all LIC levels were 2.1 ± 2.3 % and 7.2 ± 5.6 % (*t*-test: *p* < 0.001), respectively. The *D*_*TSA*_ from the OP and SA schemes of the 1D- and 2D-FCM methods for all LIC levels were 10.3 ± 9.9 % and 18.5 ± 11.2 % (*t*-test: p < 0.001), and 7.0 ± 11.9 % and 11.4 ± 14.6 % (*t*-test: p = 0.002), respectively. For both schemes, higher LIC levels increase *D*_*TSA*_ of 1D-FCM, while 2D-FCM yields a high *D*_*TSA*_ for moderate LIC levels. In contrast, MIX-FCM from both schemes can reduce *D*_*TSA*_, as compared to either 1D- or 2D-FCM alone, and yields low *D*_*TSA*_ for all LIC levels.

**Table 2:** Mean ± SD of *D*_*TSA*_ values

Scheme	Method	LIC levels			
		Mild	Moderate	Severe	All
OP	1D-FCM	0.9 ± 1.5	4.5 ± 8.1	16.9 ± 7.8	10.3 ± 9.9
	2D-FCM	5.2 ± 10.2	11.1 ± 9.0	5.9 ± 13.2	7.0 ± 11.9
	MIX-FCM	0.8 ± 1.5	2.2 ± 2.9	2.6 ± 2.1	2.1 ± 2.3
SA	1D-FCM	9.8 ± 4.7	13.3 ± 8.2	24.6 ± 10.7	18.5 ± 11.2
	2D-FCM	14.0 ± 13.7	18.4 ± 15.3	7.2 ± 13.4	11.4 ± 14.6
	MIX-FCM	9.7 ± 4.7	10.1 ± 4.8	4.9 ± 5.3	7.2 ± 5.6

Mean ± SD (%) of *D*_*TSA*_ values from1D-, 2D-, and MIX-FCM clustering by LIC level for both OP and SA schemes. MIX-FCM method delivers significantly lower error rates than either of its component parts alone

**Fig. 2:**
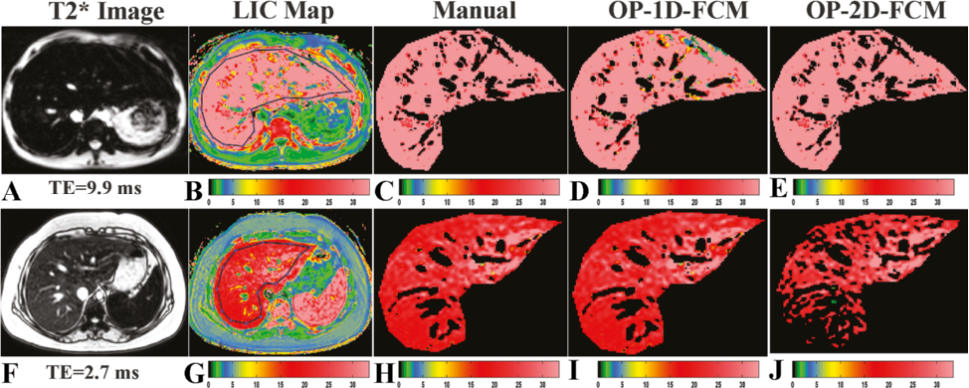
Example images from OP scheme. Example liver images, LIC, and segmentation results from two cases, with each row corresponding to a case. The first column (**a** and **f**) and second column (**b** and **g**) are theacquired image and the corresponding LIC map, respectively. Manual (**c** and **h**), OP-1D-FCM (**d** and **i**), andOP-2D-FCM (**e** and **j**) clustering results are shown in the 3rd, 4th, and 5th columns, respectively. The colormap scale bar for the LIC map is displayed below the images in mg/g dw (dry weight). 2D-FCM clusteringoutperformed 1D-FCM in the first case, with opposite performance results in the second case.

**Fig. 3:**
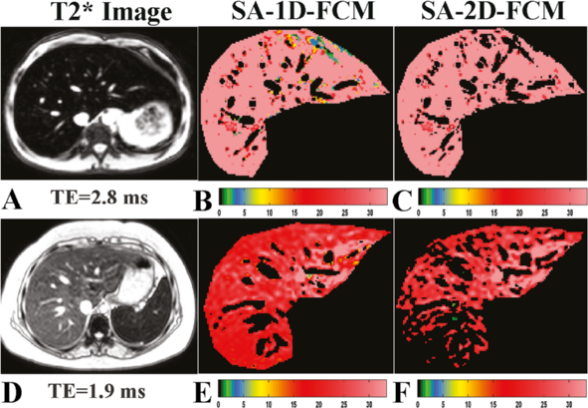
Example images from SA scheme. Example images from SA scheme using the same cases used in Fig. 2. The first column (**a** and **d**) is the acquired image while the 2nd (**b** and **e**) and 3rd (**c** and **f**) columns are theSA-1D-FCM and SA-2D-FCM clustering results, respectively.

**Fig. 4:**
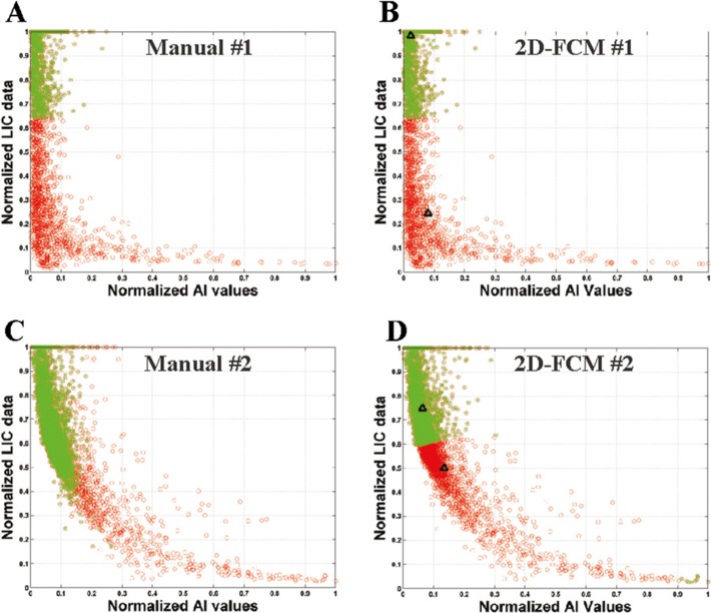
Scatter plots of normalized AI and LIC data. The scatter plots show normalized AI and LIC data from manual (*left*) and 2D-FCM (*right*) segmentations from the same cases used in Fig. 2. Empty and filled dots represent vessel and parenchyma populations, respectively, with 2D-FCM centroids denoted by *black triangles*. The 2D-FCM clustering result was similar to the manual result in the first case, but different in the second case

Figures 2 and 3 demonstrate the benefit of the MIX-FCM method from the OP and SA schemes, respectively. For both schemes, the first row shows a case for which 2D-FCM clustering provides a better segmentation result than 1D-FCM (*D*_*TSA*_: 0.05 *vs.* 28.6 % and 0.16 *vs.* 29.7 %, respectively). The second row shows the opposite result, in which *D*_*TSA*_ for 1D-FCM is better than for 2D-FCM (2.0 *vs.* 57.5 % and 8.4 *vs.* 59.3 %, respectively). Thus, the MIX-FCM method selects the 2D-FCM result for the first case (*D*_*TSA*_ = 0.05 and 0.16 %) and the 1D-FCM result for the second case (*D*_*TSA*_ = 2.0 and 8.4 %) for the OP and SA schemes, respectively. This illustrates how MIX-FCM achieves high clustering accuracy, compared with the accuracy of using each method alone. Using the cases presented in Fig. 2, Fig. 4 presents scatter plots which confirm that 2D-FCM clustering is more successful in the first case (Fig. 4b) than in the second case (Fig. 4d), with manual segmentation (Fig. 4a and c representing the first and second cases, respectively) serving as the reference. While the 2D-FCM method can yield better segmentation results than the 1D-FCM method, especially at high LIC levels, the data distribution limits clustering accuracy overall.

**Fig. 5:**
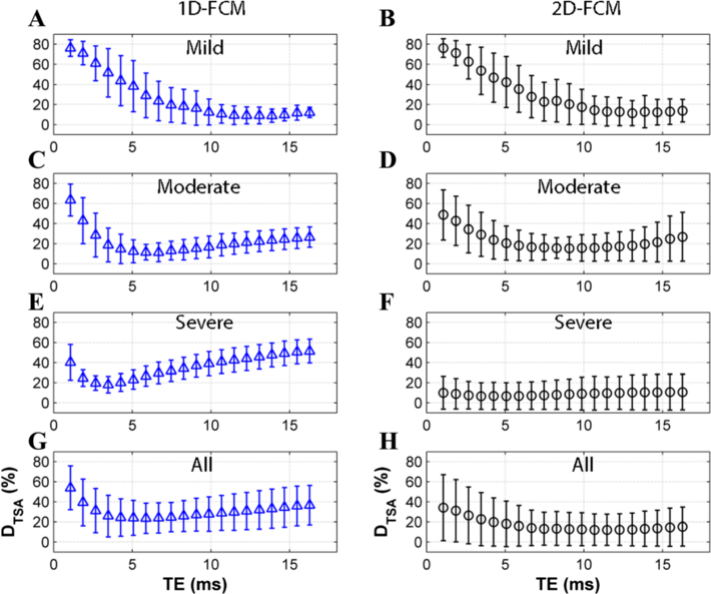
*D*_*TSA*_ as a function of TE values. Mean ± SD of *D*_*TSA*_ results from 1D- and 2D-FCM clustering for OP scheme as a function of TE values for mild, moderate, severe, and all LIC levels show that higher LIC levels correspond to lower TE-values of minimum error images

**Fig. 6:**
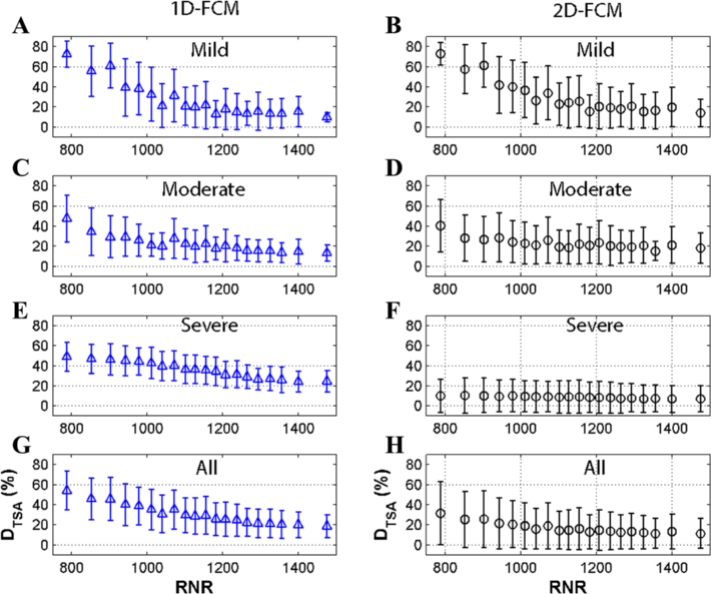
*D*_*TSA*_ as a function of RNR values. Mean ± SD of *D*_*TSA*_ results from 1D- and 2D-FCM clustering for SA scheme as a function of RNR values for mild, moderate, severe, and all LIC levels show minimum error images corresponding to highest RNR (RNR_max_) values

Figures 5 and 6 describe the segmentation errors of the FCM methods, relative to TE (OP scheme) and RNR (SA scheme), respectively. The optimal TEs, defined from the minimum *D*_*TSA*_ values, occurred on images corresponding to a TE of 5.8 and 11.5 ms for 1D- and 2D-FCM, respectively (Fig. 5g and h). However, the *D*_*TSA*_ values were not significantly different (*t*-test: *p* = NS) from the adjacent TE data. Moreover, higher LIC levels led to lower TE values, corresponding to images producing minimum *D*_*TSA*_ results (Fig. 5a-f). TEs from the OP scheme had values of 5.8 ± 4.3 and 11.5 ± 6.1 ms for 1D- and 2D-FCM, respectively. The SA scheme, on the other hand, defined the TE input from the RNR_max_ (Fig. 6). The minimum *D*_*TSA*_ values occurred at the highest RNR values for both FCM methods at all LIC levels, but the values were not significantly different (*t*-test: *p* = NS) from the neighboring RNR data. TEs from the SA scheme had a value of 8.2 ± 5.2 ms. As such, the input selection from the SA scheme should also provide minimum *D*_*TSA*_ values similar to those of the OP scheme. In this study, the mean ± SD of the TE values of the OP-MIX-FCM and SA-MIX-FCM schemes were 10.2 ± 6.3 and 8.2 ± 5.2 ms, respectively, without statistical difference (*t*-test: *p* = NS). As a result, the TE selection from the SA scheme is comparable to that of the reference scheme.

**Fig. 7:**
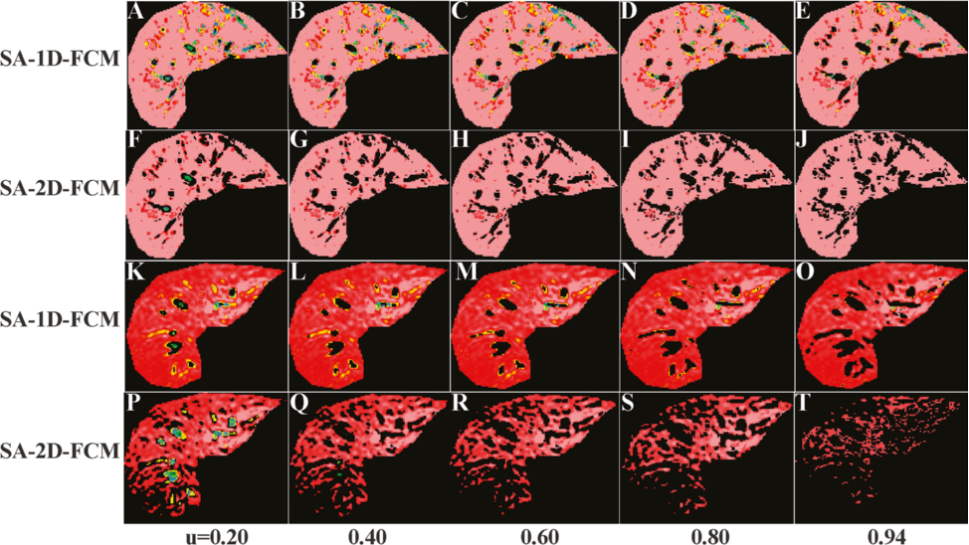
1D- and 2D-FCM images of SA scheme from various membership thresholds. 1D- and 2D-FCM images ofSA scheme from the same cases used in Fig. 2 show segmentation results from membership thresholdsof 0.20, 0.40, 0.60, 0.80, and 0.94. SA-1D-FCM (**a**-**e**) and SA-2D-FCM (**f**-**j**) clustering results of the first caseare shown in the 1st and 2nd rows, respectively, while the second case is at the 3rd (**k**-**o**) and 4th (**p**-**t**) rows, respectively. Note: User select membership threshold (u) of 0.60 on 2D-FCM image (**h**) for the first case and0.94 on the 1D-FCM image (**o**) for the second case.

The other FCM clustering variable, membership thresholds, were also investigated. Thresholds from the OP scheme had values of 0.81 ± 0.13 and 0.53 ± 0.24 for 1D- and 2D-FCM, respectively, but did not show a relationship with LIC values. Results from 1D- and 2D-FCM membership selection in the OP scheme are the same as user selection in the SA scheme. Moreover, the mean ± SD of the threshold selection from the OP- and SA-MIX-FCM schemes were comparable (0.58 ± 0.28 *vs.* 0.60 ± 0.26, respectively), implying similar selection by the reference scheme and the user. For example, Fig. 7 presents the same two cases as presented in Fig. 2, with various membership thresholds. In the first case, the user selected a result from SA-2D-FCM at 0.60 threshold (Fig. 7h), and in the second case from SA-1D-FCM at 0.94 threshold (Fig. 7o), which are the same thresholds as in the reference scheme. In summary – to obtain an optimal result, the FCM input variables, namely the TE values and the membership threshold, must be appropriately selected, in which case the SA scheme will identify input selections similar to those of the reference scheme.

LIC values reported by their medians obtained from non-segmented and segmented images were similar (17.9 ± 10.6 *vs.* 18.3 ± 10.8 mg/g dw, respectively; *t*-test: *p* = NS). As such, LIC values can withstand the outlier effect caused by vessel data. In contrast, LIC variations or the *nIQR* after excluding vessel data were significantly lower by around 30 % (16.4 ± 7.9 % (segmented) *vs.* 23.2 ± 9.2 % (non-segmented); *U* test: *p* < 0.001) (Table 3). The *nIQR* reductions from replacing non-segmented images with segmented images were approximately 14 % (14.4 ± 4.6 *vs.* 16.8 ± 4.8 %; *U* test: *p* = 0.0330) at the mild LIC level, 16 % (18.8 ± 5.4 % *vs.* 22.4 ± 8.1 %; *U* test: *p* = 0.0205) at the moderate LIC level, and 38 % (16.2 ± 9.6 % *vs.* 26.3 ± 9.7 %; *U* test: *p* < 0.001) at the severe LIC level. Put another way, inclusion of vessel data in the LIC calculation will overestimate the distribution of iron in liver parenchyma by almost 38 % at the severe overload range. The exclusion of vessels can, therefore, further improve assessment of LIC measurement. Although all three of the FCM clustering methods from the OP scheme resulted in similar *nIQR* values when compared with the manual method, their CVs of *nIQR* were markedly different. 1D-FCM had the highest CV (14.9 %), compared with CVs of 5.2 and 2.4 % for the 2D- and MIX-FCM methods, respectively. However, both 1D- and 2D-FCM methods suffer from high CVs at the severe overload range (21.0 and 6.7 %, respectively). The SA-MIX-FCM scheme achieved an acceptable CV (4.1 %), as compared to reference (2.4 %). Overall, MIX-FCM clustering of both schemes yielded results that match the reference (manual segmentation) to a suitable degree, while individual results from 1D- and 2D-FCM clustering were found to be less accurate and less reliable, especially at the severe overload range.

**Table 3:** Mean ± SD of *nIQR* values

Type	*nIQR* (%)			
	Mild	Moderate	Severe	All
None	16.8 ± 4.8	22.4 ± 8.1	26.3 ± 9.7	23.2 ± 9.2
Manual	14.4 ± 4.6	18.8 ± 5.4	16.2 ± 9.6	16.4 ± 7.9
OP-MIX-FCM	14.4 ± 4.5	18.8 ± 5.3	16.2 ± 9.5	16.4 ± 7.8
SA-MIX-FCM	14.4 ± 4.5	18.8 ± 5.4	16.2 ± 9.6	16.4 ± 7.9

Mean ± SD of *nIQR* values obtained for non-segmented images and images segmented by manual, OP-MIX-FCM, and SA-MIX-FCM schemes. Results are tabulated by LIC level and demonstrate that all segmentation types yield similar variations (*nIQR*), all of which are substantially lower than those corresponding to non-segmented images

In summary, as compared with the basic (1D-FCM) and proposed (2D-FCM) methods, the OP-MIX-FCM scheme yielded a significantly (*t*-test: *p* < 0.001) lower segmentation error (10.3 ± 9.9 % and 7.0 ± 11.9 *vs.* 2.1 ± 2.3 %, respectively) and CV of *nIQR* (14.9 % and 5.2 *vs.* 2.4 %, respectively). The SA-MIX-FCM scheme delivered an outcome (segmentation error: 7.2 ± 5.6 %; CV of *nIQR*: 4.1 %) comparable to that of the reference OP-MIX-FCM scheme.

## Discussion

Typically, calculated LIC values from the entire liver ROI include some vessel data, but reporting by their medians can reduce outlier data caused by the vessel parts [5, 15, 17, 22]. Their IQRs, on the other hand, are overestimated due to this vessel data (especially at the severe LIC range) [17], which reduces the reliability and precision of the measurement. Segmentation of the vessels increases measurement precision yielding only iron distribution in the liver parenchyma, but manual segmentation is time consuming and introduces user dependencies, rendering it unsuitable and impractical in a clinical setting.

In this study, we propose a new FCM (2D-FCM) algorithm based on a combination of AI and LIC values for vessel segmentation. We found that it effectively improves segmentation accuracy, as compared with conventional FCM (1D-FCM), at the severe overload range. It is, however, less accurate at the other ranges. The 2D-FCM method is, therefore, not entirely superior to 1D-FCM. Alternatively, we propose the MIX-FCM method, which showed ability to substantially improve segmentation accuracy with lower CV of *nIQR* at all iron overload ranges. This study also investigated a semi-automatic (SA-MIX-FCM) scheme that can be practically implemented for clinical application and that provides a segmentation outcome comparable to that of the reference (OP-MIX-FCM).

In this study, the 1D-FCM method from the OP scheme had the highest error (17 %) at the severe overload range. This result eventuated from the optimal (the minimum *D*_*TSA*_) clustering parameters (i.e., the optimal TE image and membership threshold), and yet still yielded very poor performance. This result is likely explained by the bias field and artifacts that are detrimental to intensity-based classification methods, such as FCM [23–25]. Instead of using gain field correction to improve performance, as reported in previous studies [17, 27, 28], we performed 2D-FCM, which resulted in 7 % segmentation error (OP scheme). 2D-FCM segmentation, as compared with 1D-FCM, was found to be more accurate at the severe overload range and less accurate at the other two ranges (Table 2). The improvement is the result of the high contrast between the parenchyma and vessels in the LIC data (Fig. 2e), which can reduce the bias field effect of the AI values. In contrast, 2D-FCM is less effective at the other two ranges due to low contrast between the two anatomical classes in the LIC data. Additionally, there are some cases in which 2D-FCM clustering results with very high segmentation errors (more than 30 %) are likely caused by a non-spherical shape and a population concentration of AI values and LIC map (Figs. 2j and 4d). This population characteristic renders 2D-FCM clustering less effective, because the algorithm defines the classification based on the minimum Euclidean (spherical) distance between each data point and the centroid of the cluster groups [23, 24]. At the severe level, the high contrast of LIC data can minimize such a non-spherical effect (Figs. 2e and 4b). On its own, the 2D-FCM algorithm is not always preferable to the standard 1D-FCM method.

In our study, using a fixed TE and membership threshold input as suggested by Positano, et al. [17] did not produce an optimal clustering result. Their report suggested using the last TE image and a threshold of 0.5 for FCM clustering to obtain segmentation errors of approximately 50 and 30 % for input data without and with gain field correction, respectively. The advice to use the last TE image holds true only in the mild overload range, which has the lowest error around that TE (Fig. 5a and b). For the moderate and severe overload ranges, however, this image choice does not provide optimal results, because minimum error can be acquired from an earlier TE image (Fig. 5c-f). Regarding membership threshold, a fixed threshold of 0.5 is reasonable for 2D-FCM (0.53), but too low for 1D-FCM (0.81); so, a variable threshold should provide better clustering results than a fixed threshold. In our study, if the same fixed TE and threshold were applied, the error would have been 55 and 25 % (data not shown) for the 1D-FCM and 2D-FCM, respectively. This error rate is substantially higher than the best outcomes from the OP scheme of 10.3 and 7 %, respectively.

From our study, the optimal combined scheme (OP-MIX-FCM) yielded a classification error of only about 2 %. This low error rate was attributed to the complementary effect of the 1D- and 2D-FCM methods, in which the latter method reduces the bias field effect, thus impairing the former method at the severe overload range, while the detrimental effect of the non-spherical shape population and low contrast in the LIC data at the other two ranges is mitigated by using 1D-FCM results. As a result, MIX-FCM is vastly more practical in a clinical setting, as compared to the use of each method alone.

Our proposed practical implementation (SA scheme) of the MIX-FCM method is based on a finding from the optimal FCM input parameters, which showed an inverse relationship between optimal TE images and LIC values. This finding was then integrated into the SA scheme to automatically select the TE image input based on RNR_max_ value. Membership threshold, on the other hand, did not show any such clear relationship, so user selection continues to be required. The SA scheme does fit the criteria for a practical implementation, because it only requires the user to select appropriated membership thresholds from both the 1D- and 2D-FCM methods and then chose the desired output (the SA-MIX-FCM). We also found that the SA-MIX-FCM scheme can achieve segmentation accuracy comparable to that of OP-MIX-FCM.

We also found that LIC values calculated from non-segmented and segmented images to be statistically insignificant, which confirms the benefit of reporting LICs by their median, as found in previous studies [5, 15, 17, 22]. The *nIQR*s from former images, in contrast, are significant higher. This holds true, especially at the severe overload range, due to the considerable difference in LIC values between vessel and parenchyma. Exclusion of such vessels as in the later images, therefore, can help to improve precision of measurement, which relates only to iron distribution from the liver parenchyma. This should improve assessment of LIC measurement in both longitudinal studies and clinical trials.

This study was limited by the following factors and circumstances. First, due to the generally young age of our thalassemia patients, no diffuse or focal liver diseases were observed in our study. Inclusion of these pathologies would very likely alter our FCM clustering results. Further studies are needed to investigate the effects of comorbidities on FCM clustering results. Next, FCM clustering errors may be underestimated due to bias resulting from manual segmentation, which was performed by only one expert who was also familiar with the FCM clustering technique. A blinded experiment should be performed to minimize potential bias. Finally, we only investigated a standard FCM algorithm without gain field correction, because the standard routine already existed in the MATLAB commercial software that we used for analysis and we aimed for a simple implementation that could be generally applied in a clinical setting. Nevertheless, our 1D- and 2D-FCM clustering results using the same FCM variables are comparable to 1D-FCM results without and with gain field correction reported from another study [17]. Accordingly, additional study should focus on testing our SA scheme in a large number of cases in routine clinical usage and, further, investigate its intra- and inter-observer variability. Our SA scheme should also be further investigated regarding automatic selection criteria for TE image input. Such an investigation may reduce segmentation error to a level similar to that of the optimal (OP) scheme.

## Conclusions

Our proposed 2D-FCM clustering method, as compared with the 1D-FCM method, improves accuracy of segmentation only at the severe overload range. Our proposed MIX-FCM scheme, however, substantially improves segmentation accuracy at all ranges and has the lowest CV of *nIQR* among all methods. The proposed SA-MIX-FCM scheme can be practically implemented in a routine clinical setting and provides a segmentation outcome comparable to the reference (OP-MIX-FCM) scheme. Implementation should reasonably be expected to improve LIC assessments, especially at the severe overload range, and would benefit thalassemia patients, particularly with regard to monitoring efficacy of the chelator.

## Abbreviation

AI: Acquired Image
C-EXP: Constant-offset mono-EXPonential model
*D*_*TSA*_: Segmentation Error from both vessel and parenchyma clusters
FCM: Fuzzy C-Mean
IQR: Inter-Quartile Range
LIC: Liver Iron Concentration
MRI: Magnetic Resonance Imaging
*nIQR*: Normalized Interquartile Range
OP: Optimal scheme
RNR: Range to Noise Ratio
RNR_max_: Maximum of Range to Noise Ratio
ROI: Region of Interest
SA: Semi-Automatic scheme
*TSA*: Tissue Segmentation Accuracy
